# Vitamin D status and risk of non-Hodgkin lymphoma: An updated meta-analysis

**DOI:** 10.1371/journal.pone.0216284

**Published:** 2019-04-29

**Authors:** Hye Yin Park, Yun-Chul Hong, Kyoungho Lee, Jaewoo Koh

**Affiliations:** 1 Samsung Health Research Institute, Samsung Electronics Co. Ltd., Hwaseong-si, Gyeonggi-do, Korea; 2 Institute of Environmental Medicine, Seoul National University Medical Research Center, Seoul, Korea; 3 Department of Preventive Medicine, Seoul National University College of Medicine, Seoul, Korea; 4 Environment Health Center, Seoul National University, Seoul, Korea; Universita degli Studi di Brescia, ITALY

## Abstract

**Purpose:**

This meta-analysis aimed to extensively investigate the association between various measures of vitamin D status and non-Hodgkin lymphoma (NHL) and its subtypes.

**Methods:**

We searched MEDLINE (PubMed), Embase, and the Cochrane Library in February 2018. Two authors independently reviewed and selected articles based on predetermined criteria.

**Results:**

A total of 30 studies with 56,458 NHL cases were finally selected, with 24, 9, and 3 studies on sunlight/ultraviolet radiation (UVR) exposure, dietary intake, and serum/plasma 25-hydroxyvitamin D levels, respectively. Significant protective effects of overall sunlight/UVR exposure on NHL and subtypes were observed, with summary relative risks (RRs) ranging from 0.67–0.80 (RR for NHL = 0.80; 95% confidence interval [CI]: 0.71–0.90) among subjects with high exposure compared to those with low exposure. The results were consistent with various classifications of sunlight/UVR exposure. In contrast, when exposure measures of dietary vitamin D intake (RR for NHL = 1.03; 95% CI: 0.90–1.19) and serum/plasma 25-hydroxyvitamin D levels (RR for NHL = 0.97; 95% CI: 0.82–1.15) were used, risk estimates were inconsistent or non-significant for NHL and the subtypes.

**Conclusion:**

While risk estimates varied by different measures of vitamin D status, a protective effect of sunlight/UVR exposure on NHL incidence was verified, across most of the tested subtypes as well as exposure categories.

## Introduction

Non-Hodgkin lymphoma (NHL) is one of the most common hematologic malignancies, accounting for 3% of all incident cancer cases according to GLOBOCAN reports [1]. There has been a substantial increase in NHL incidence rates over the last decade with a marked increase in the number of less-frequently investigated races/ethnicities, which in turn emphasizes the role of environmental factors and demands continued epidemiological research [2–4]. One of the environmental factors receiving attention is vitamin D, especially since vitamin D deficiency has currently become pandemic [5].

Previous studies have investigated the influence of vitamin D status on NHL with exposure measures using sunlight or ambient ultraviolet radiation (UVR) exposure, dietary vitamin D intake, and 25-hydroxyvitamin D (25(OH)D) levels. Pooled analysis results from the International Lymphoma Epidemiology Consortium (InterLymph; https://epi.grants.cancer.gov/InterLymph/) have shown the protective effect of recreational/total sunlight exposure, or in composite measures, on the incidence of NHL [6] and its B- or T-cell subtypes, including diffuse large B-cell lymphoma (DLBL) [7], follicular lymphoma (FL) [8], chronic lymphocytic leukemia and small lymphocytic lymphoma (CLL/SLL) [9], marginal zone lymphoma (MZL) [10], mantle cell lymphoma [11], and peripheral T-cell lymphomas (PTCL) [12]. A separate meta-analysis found an insignificant association of NHL incidence with dietary vitamin D and 25(OH)D levels [13].

Based on progress in the classification and diagnosis of NHL [14,15], and persistent emphasis on the vitamin D-NHL association [16], we conducted a meta-analysis of case-control and cohort studies to ascertain the association between vitamin D status and the risk of NHL and all traceable subtypes. Vitamin D status was classified by sunlight/UVR exposure, dietary vitamin D intake, and serum/plasma 25(OH)D level.

## Materials and methods

### Literature search

Using PubMed, Embase, and the Cochrane Library, literature published until February 2018 were searched using the following terms: “vitamin D”, “25-hydroxyvitamin D”, “sun exposure”, “ultraviolet radiation”, “UV-B radiation”, “solar radiation”, and “non-Hodgkin lymphoma”, “diffuse large B-cell lymphoma”, “follicular lymphoma”, “chronic lymphocytic leukemia”, “small lymphocytic lymphoma”, “mantle cell lymphoma”, “marginal zone lymphoma”, “T-cell lymphoma”, and “peripheral T-cell lymphoma”. In addition to keyword search, reference lists of all relevant articles were manually searched to discover additional studies.

### Selection criteria

Studies included in the current meta-analysis met the following criteria: (1) title and abstract, at least, were provided in English language; (2) study was an original article, either case-control or cohort study, with outcomes measured as odds ratios (ORs), relative risks (RRs), or hazard ratios (HRs) and their 95% confidence intervals (CIs); (3) incidence of NHL or NHL subtypes identified by clinical diagnosis or connected registries; (4) indices of vitamin D status included sunlight/UVR exposure, dietary vitamin D intake, and serum or plasma 25(OH)D. All literature search and selection processes were cross-checked by two researchers (HYP and JK).

### Selection of relevant studies

Although one nested case-control study [17] with outcome measurement in incidence rate ratio did not meet the selection criteria, it was included in the analysis due to its design and statistical methods, and the results were interpretable as RRs.

If overlapping studies that included the same study population and methods were found, literature in the most recent publication or with the largest sample or population size were selected. For instance, the InterLymph Consortium collaborated with many of the existing studies to perform a pooled analysis [18] and many of the existing data and results had been integrated; thus, it was necessary to distinguish the study population, period, and particular exposure/outcome measure of each relevant study.

Studies were finally selected for the meta-analysis and evaluated for quality reporting standards using the Newcastle-Ottawa Scale (NOS) [19].

### Main and subgroup analysis

The association between vitamin D status and NHL was assessed using varied combinations of exposure and outcome measures. Measures of sunlight/UVR exposure among the reviewed articles were obtained from questionnaire responses and/or estimated using geographical conditions, e.g., estimated ground-level UVR exposure from the Total Ozone Mapping Spectrometer dataset of the National Aeronautics and Space Administration. The different exposure units and their ranges were assessed to produce a single integrated measure for main association evaluation. Separable sunlight/UVR exposure measures were also grouped into the following categories: (i) ‘sunburn’, (ii) ‘bathing vacation’, (iii) ‘artificial tanning’, (iv) ‘sunbath/suntan’, (v) ‘ambient exposure (e.g. Total Ozone Mapping Spectrometer data)’, (vi) ‘outdoor/recreational activity’, (vii) ‘total exposure (composite, or h/wk)’, and (viii) ‘recreational exposure (composite)’. The latter two assessment variables with “composite” categories were borrowed from the InterLymph literature [7–10]. In the main meta-analysis with overall sunlight/UVR exposure as the independent variable, if multiple exposure measures were present in a single study, categories in order of priority for selection were as follows: ‘sunbath/suntan’, ‘bathing vacation’, ‘sunburn’ or ‘ambient exposure’, ‘outdoor/recreational activity’ or ‘composite recreational exposure’, and ‘artificial tanning’ or ‘composite total exposure’. If information on lifetime exposure period was unavailable, an exposure period of 10–30 s was adopted.

While all studies with dietary vitamin D intake were interpretable to a single unit (IU/day), comparison of exposure categories was carried out in two ways, as approximately 200 IU/day or maximum intake versus the lower baseline reference. The intake level of 200 IU/day was selected due to the availability of the categorical criteria values.

The reference range for serum/plasma 25(OH)D level was set at 50–75 nmol/L, and due to data availability, estimates with 25(OH)D levels below the reference range were extracted instead of those with higher levels. Outcome assessment was considered for the association between 25(OH)D level and NHL, where only two and one studies with NHL and B-cell NHL, respectively, were available. Thus, all three results were used in the meta-analysis, as previously reported [13].

### Statistical analysis

From the selected case-control and cohort studies, ORs, RRs, or HRs and their 95% CIs were used to calculate the summary RR. Statistical heterogeneity among the studies was tested using the Cochran Q and *I*^2^ statistics, with p-value 0.10 for the former and a value of 50% for the latter considered as significant levels for heterogeneity. Fixed- or random-effect models were used if the meta-analysis did not or did show heterogeneity, respectively.

In the meta-analysis testing the association between sunlight/UVR exposure and NHL, several subgroup analyses were carried out based on various study features, i.e., study design (cohort or case-control studies), population composition (Caucasian or non-Caucasian, general population, or a particular group), and quality standard (NOS score ≥7 or <7). Studies that significantly changed heterogeneity were also tested in sensitivity analyses. The study design was also separately tested in association analysis on dietary intake and NHL.

Begg’s and Egger’s tests were used to test for publication bias in meta-analyses with p-values at 0.05 considered as significant. All statistical analyses were performed using STATA version 12 (Stata Corp., College Station, Texas, USA).

## Results

### Identification of relevant studies

**Fig 1:**
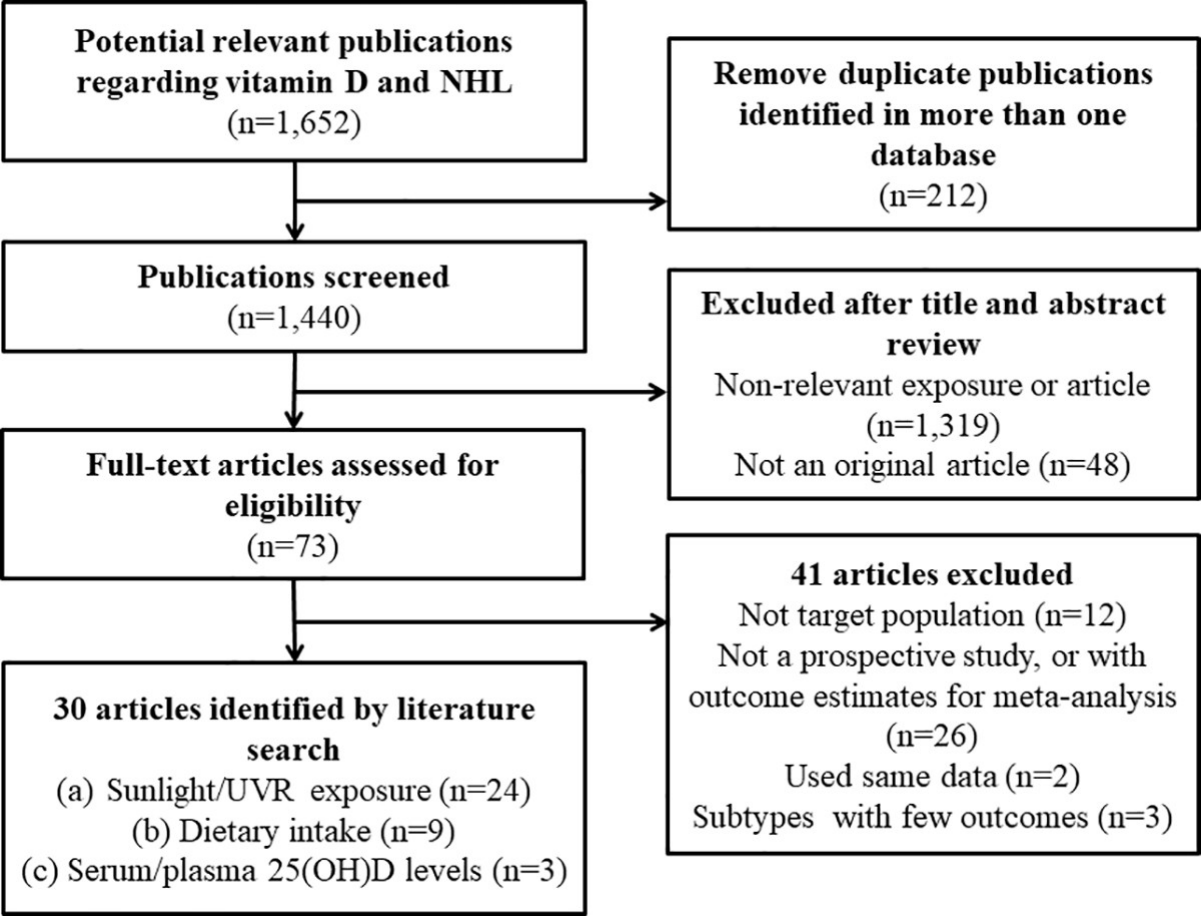
Flow diagram of identification of relevant studies.

Of 1,652 articles included based on the search strategy, 212 duplicates in more than one database were identified and removed (Fig 1). After screening for title and abstract, 1,319 articles deemed to be non-relevant to the exposure or outcome of interest were also removed (e.g., skin phototherapy and prognosis in existent NHL). A full-text assessment was performed on the remaining 121 articles; 48 were removed as they were not original studies (e.g., reviews). Further, we excluded 12 articles as they did not address the general adult population (e.g., children or workers occupationally exposed to UVR) and 26 articles which were not prospective studies or did not assess the outcome measures of interest (e.g., standardized incidence rate). Two more articles were removed due to duplicate data source and exposure-outcome association results. Finally, 3 articles which each assessed a single NHL subtype (e.g., mantle cell lymphoma) were also eliminated.

Of the remaining 30 studies [6–10,17,20–43], the association results between NHL or NHL subtypes and vitamin D exposure measures, i.e., sunlight/UVR exposure, dietary intake, and serum/plasma 25(OH)D levels, were extracted from 24, 9, and 3 studies, respectively.

Of the remaining 30 studies [6–10,17,20–43], 24, 9 and 3 studies were used to extract association results between NHL or NHL subtypes and vitamin D exposure measures, i.e. sun/UVR exposure, dietary intake, and serum/plasma 25-hydroxyvitamin D levels, respectively.

### Characteristics of selected studies

A detailed description of the characteristics of selected studies is shown in Table 1 (see also S1 Table). A total of 56,458 cases were included in the analysis. All study subjects were over 17 years old, and most studies included subjects of both genders except for four studies that included females only [22–24,26]. Most participants were Caucasians residing in North America and Europe, except for two studies conducted in Singapore and the Middle East [41,42], and were mostly general, non-specific populations except for four studies on nurses [23,26], teachers [21], and radiation technologists [24]. Baseline enrolment in cohort studies was carried out from 1976–2001, and follow-up was conducted from 1996–2010. Case collection for case-control studies was conducted during 1974–2010. All studies were matched for or adjusted for age and gender, as well as race/ethnicity for multi-ethnic populations, region/center in multi-center studies, and total energy intake in studies on dietary vitamin D intake. The NOS of all studies ranged from 6 to 9 (S1 and S2 Tables).

**Table 1:** General characteristics of the studies on vitamin D status and non-Hodgkin lymphoma risk.

Author	Ref. no.	Type	Study	Country	Recruit period	Case no.	Control/ pop. no.	Exposure*	Outcome	Matched/adjusted variables
Erber et al., 2010	[20]	cohort	MEC (multiethnic cohort)^1)^	USA	1993–1996	male 514, female 425	193,050	diet	NHL, DLBL, FL, CLL/SLL	education, body mass index, alcohol intake, total energy intake (plus race/ethnicity)
Freedman et al., 2010	[21]	cohort	USRT study	USA	1983–2005	137	64,103	sunlight/UVR (v, vii)	NHL	age, sex, race/ethnicity
Veierød et al., 2010	[22]	cohort	Norwegian-Swedish Women's Lifestyle and Health Cohort Study	Sweden, Norway	1991–1992	158	104,953	sunlight/UVR (i, ii, iii)	NHL	age, region of residence, eye color, hair color, skin reaction after heavy sun exposure in the beginning of the summer and after repeated sun exposure (plus solar exposure in artificial tanning)
Bertrand et al., 2011	[23]	cohort	NHS (Nurses' Health Study)^1)^	USA	1976–2006	808	115,482	sunlight/UVR (v); diet	NHL, DLBL, FL, CLL/SLL (dietary: only NHL)	age, smoking, body mass index, height
Chang et al., 2011	[24]	cohort	CTS (California Teachers Study)	USA	1995–1996	629	121,216	sunlight/UVR (v); diet	NHL, DLBL, FL, CLL/SLL (dietary: only NHL)	age, calendar-year effect
Lin et al., 2012	[25]	cohort	NIH-AARP Diet and Health Study	USA	1995–1996	NHL 2,731, DLBL 1,059, FL 577, CLL/SLL 237, T-cell 188	450,934	sunlight/UVR (v)	NHL, DLBL, FL, CLL/SLL, T-cell	age at baseline, sex, BMI, caloric intake, intake of fruit, vegetables, red and white meat, alcohol consumption, tobacco smoking, education, physical activity, median household income
Zhang et al., 2013	[26]	cohort	NHS II	USA	1989–2009	185	73,358	sunlight/UVR (iii)	NHL	age,body mass index, alcohol, physical activity, multivitamin, smoking, oral contraceptive use, menopausal status, hormone replacement therapy use, outdoor sun exposure during high school/college, at 25-35years, UV index of residence, dietary & supplementary vitamin D intake
Hughes et al., 2004	[27]	case-control	(NSW)^2)^	Australia	2000–2001	704	694	sunlight/UVR (ii)	NHL	age, sex, state of residence at diagnosis, ethnicity, skin color, ability to tan
Smedby et al., 2005	[28]	case-control	SCALE (Scandinavian lymphoma etiology)^3)^	Denmark, Sweden	1999–2002	3,055	3,187	sunlight/UVR (i, ii, iii, iv)	NHL, DLBL, FL, CLL, T-cell	age, sex, country, skin type
Chang et al., 2006	[29]	case-control	SCALE (Scandinavian lymphoma etiology)^2)^	Sweden	2000–2002	591	460	diet	NHL, DLBL, FL, CLL, T-cell	age, sex, total energy intake, intake of retinol/vitamin D/calcium/phosphorus
Hartge et al., 2006	[30]	case-control	(SEER)^2)^	USA	1998–2002	551	462	sunlight/UVR (i, iii, iv, v); diet	NHL	age, sex, race/ethnicity, study region, exercise, total energy intake
Polesel et al., 2006	[31]	case-control	(Aviano-Naples)^2)^	Italy	1999–2002	190	484	diet	NHL, DLBL, FL	age, sex, center, education, place of birth, HCV test, total energy intake
Soni et al., 2007	[32]	case-control	(Nebraska)^3)^	USA	1999–2002	387	535	sunlight/UVR (v); diet	NHL, DLBL, FL, CLL/SLL, MZL, B-/T-cell	age, sex, family history of cancer
Weihkopf et al., 2007	[33]	case-control		Germany	1999–2003	589	589	sunlight/UVR (ii, iv, vi)	DLBL, FL, CLL/SLL, MZL, B-/T-cell	age, sex, region, smoking status, alcohol consumption
Zhang et al., 2007	[34]	case-control	(Yale)^3)^	USA	1996–2000	601	717	sunlight/UVR (i, ii, iv)	NHL, DLBL, FL, CLL/SLL, MZL, B-/T-cell	age, race/ethnicity, family history of NHL, education, eye color, skin type
Boffetta et al., 2008	[35]	case-control	Epilymph^3)^	France, Germany, Ireland, Italy, Spain	1998–2004	1,518	2,124	sunlight/UVR (iii, v)	NHL, DLBL, FL, CLL/SLL, B-cell, T-cell	age, sex, region(center), education, skin reaction to sun, questionnaire type
Grandin et al., 2008	[36]	case-control	Engela (France)^2)^	France	2000–2004	395	698	sunlight/UVR (iii, vi)	NHL	age, sex, region(center), outdoor activity frequency, artificial radiation/outdoor activity (stratified)
Kricker et al., 2008	[6]	case-control	InterLymph (older)^3)^	N.America, Europe, Australia	1995–2005	8,243	9,697	sunlight/UVR (vii, viii)	NHL, DLBL, FL, CLL/SLL, MZL, B-/T-cell	age, sex, race/ethnicity, study
Kelly et al., 2010	[37]	case-control	(Univ. of Rochester)^2)^	USA	2005–2007	129	139	sunlight/UVR (i, iii, iv, vii); 25(OH)D	NHL	sunlight/UVR: age, sex, race/ethnicity, skin cancer diagnosis, family history of lymphoma & other cancer, body mass index, alcohol, sun exposure variables (eg. sunburn, tanning) 25(OH)D: age, sex, race/ethnicity, prior skin cancer diagnosis, family history of lymphoma and other cancer, body mass index, season
Purdue et al., 2010	[38]	case-control	VDPP (Vitamin D Pooling Project of Rarer Caners; ATBC, CPS-II, MEC, NHS, NYU-WHS, PLCO, SMHS, SWHS	USA, Finland, China	1974–2008	1,353	1,778	25(OH)D	NHL, DLBL, FL, CLL/SLL	age at blood collection, sex, race/ethnicity, date of blood draw, height (plus menopausal status)
Kelly et al., 2012	[39]	case-control	(Mayo Clinic)^3)^	USA	2002–2008	1,009	1,233	sunlight/UVR (vii)	NHL, DLBL, FL, CLL/SLL	age, sex, region, family history of lymphoma
Mikhak et al., 2012	[40]	case-control	(UCSF-II)^2)^	USA	2001–2006	2,052	2,081	diet	NHL, DLBL, FL, CLL/SLL, MZL, T-cell	age, sex, county, total energy intake
Wong et al., 2012	[41]	case-control		Singapore	2004–2008	465	830	sunlight/UVR (vi)	NHL, B-/T-cell	age, sex, region(center), month of diagnosis, ethnicity, skin color, education, housing type, body mass index, history of any cancer in 1st-degree relatives
Łuczyńska et al., 2013	[17]	case-control	EPIC (European Prospective Investigation into Cancer and Nutrition)	Denmark, Italy, Netherlands, Norway, Spain, Sweden, UK, Germany, Greece	1992–2000	1,127	1,127	diet; 25(OH)D	DLBL, FL, CLL, B-cell	age, sex, region(center), follow-up length, time and date of blood collection, smoking status, alcohol consumption at baseline, education, BMI, physical education, total energy and calcium intake
Cerhan et al., 2014	[7]	case-control	InterLymph	N.America, Europe, Australia	1976–2008	4,667	22,639	sunlight/UVR (viii)	DLBL	age, sex, race/ethnicity, study
Linet et al., 2014	[8]	case-control	InterLymph	N.America, Europe, Australia	1976–2008	3,530	22,639	sunlight/UVR (vii, viii)	FL	age, sex, race/ethnicity, study
Slager et al., 2014	[9]	case-control	InterLymph	N.America, Europe, Australia	1976–2008	2,440	15,186	sunlight/UVR (vii, viii)	CLL/SLL	age, sex, race/ethnicity, study
Bracci PM et al., 2014	[10]	case-control	InterLymph	N.America, Europe, Australia	1976–2008	13,766	1,052	sunlight/UVR (viii)	MZL	age, sex, race/ethnicity, study
Kleinstern et al., 2017	[42]	case-control		Israel, Palestine	2010–2014	823	808	sunlight/UVR (vi)	DLBL, FL, B-cell	age, sex, marital status, education, ethnic origin, residential region
Wang et al., 2017	[43]	case-control	LA County NHL Case-Control Study	USA	2004–2008	625	625	sunlight/UVR (i, iii)	DLBL, FL, CLL/SLL, MZL, B-cell	age, race/ethnicity, socioeconomic status, standard error of skewness, family history of cancer

Abbreviations: NHL, non-Hodgkin lymphoma; DLBL, diffuse large B-cell lymphoma; FL, follicular lymphoma; CLL/SLL, chronic lymphocytic leukemia and small lymphocytic lymphoma, MZL, marginal zone lymphoma.

^1)^ Later integrated to VDPP study; exposure-outcome measures do not overlap in the current study

^2, 3)^ Later integrated to InterLymph study; exposure-outcome measures do not overlap (2) or partly overlap (3) in the current study

*underlined exposure measures were selected in the main analysis as the overall sunlight/UVR exposure indices: (i) sunburn; (ii) bathing vacation; (iii) artificial tanning; (iv) sunbath/suntan; (v) ambient exposure; (vi) outdoor/recreational activity; (vii) total exposure; (viii) recreational exposure

### Vitamin D status and the incidence of NHL and subtypes

**Fig 2:**
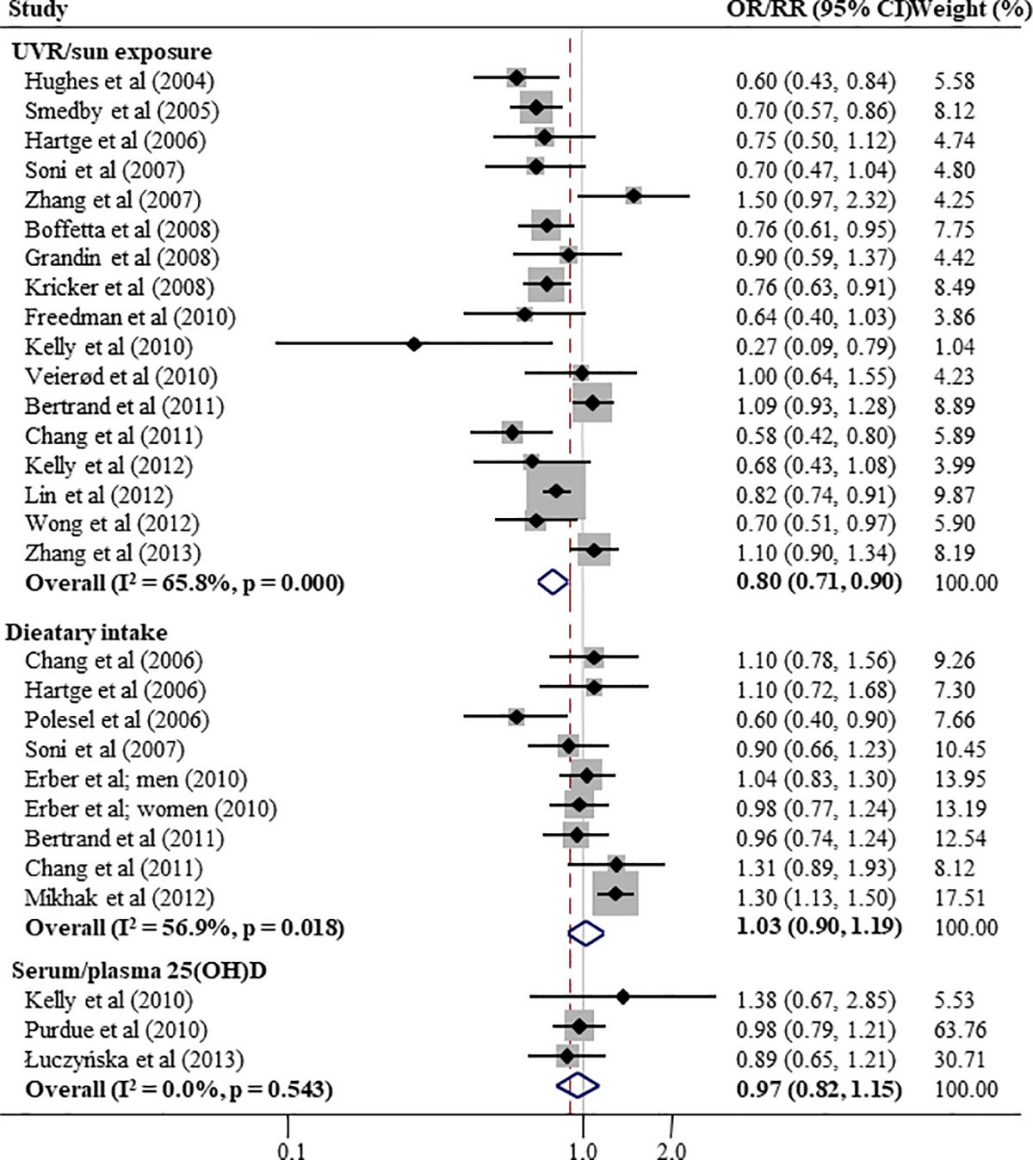
Meta-analysis of the association between vitamin D status and non-Hodgkin lymphoma incidence by different sources of exposure. (a) overall sunlight/UVR exposure; (b) dietary vitamin D intake; (c) serum/plasma 25-hydroxyvitamin D. Meta-analyses results are presented in summary relative risks (RR) and their 95% confidence intervals (95% CI).

Risk estimates for sunlight/UVR exposure, dietary intake, and 25(OH)D levels on NHL and its subtypes are shown in Table 2 and Fig 2. A significant protective association between sunlight/UVR exposure and the risk of NHL was observed, with RRs ranging from 0.67–0.80 (RR = 0.80, 95% CI: 0.71–0.90 for all evaluated studies, and RR = 0.86, 95% CI 0.79–0.94 with the maximum number of included studies) among subjects with high exposure to sunlight/UVR radiation compared to those with low exposure. When stratified by study types, statistical significance remained only in the 11 case-control studies (RR = 0.75, 95% CI: 0.68–0.82), but not in the six cohort studies (RR = 0.88, 95% CI: 0.72–1.06). When the meta-analyses were confined to studies with NOS ≥7 or the general population (i.e., excluding studies on nurses, teachers, and radiation technologists), significant associations were also found (RR = 0.80, 95% CI: 0.75–0.86 and RR = 0.77, 95% CI: 0.72–0.82, respectively). Significant associations were also observed for the subtypes DLBL (RR = 0.72, 95% CI: 0.54–0.97), FL (RR = 0.81, 95% CI: 0.73–0.90), and MZL (RR = 0.70, 95% CI: 0.57–0.87), but not for CLL/SLL (RR = 0.87, 95% CI: 0.68–1.11), B-cell NHL (RR = 0.84, 95% CI: 0.68–1.05), and T-cell NHL (RR = 0.70, 95% CI: 0.48–1.01). In a meta-regression carried out to further assess for heterogeneity in the association between sunlight/UVR exposure and NHL incidence, all tested factors showed non-significance except for year of study commencement (RR = 0.98, 95% CI = 0.97–0.995; S3 Table and S2 Fig).

**Table 2:** Risk estimates on vitamin D status and non-Hodgkin lymphoma risk by different sources of exposure.

		Summary RR (95% CI)	Study no.	I^2^	p-hetero	Begg's test	Egger's test
***(a) Sunlight/UVR exposure***							
NHL	All	0.80 (0.71–0.90)	17	65.8%	0.000.	0.387	0.259
	Cohort study	0.88 (0.72–1.06)	6	76.6%	0.001	0.707	0.793
	Case-control study	0.75 (0.68–0.82)	11	38.9%	0.090	1.000	0.835
	NOS ≥7	0.80 (0.75–0.86)	10	49.2%	0.039	0.283	0.407
	General population	0.77 (0.72–0.82)	14	42.1%	0.048	1.000	0.450
DLBL	All	0.72 (0.54–0.97)	7	80.7%	0.000.	0.23	0.313
	Exc. Kleinstern [42]	0.67 (0.53–0.84)	6	58.4%	0.035	0.06	0.031
FL	All	0.81 (0.73–0.90)	8	48.3%	0.060	0.711	0.574
	Exc. Kleinstern [42]	0.78 (0.70–0.88)	7	26.7%	0.225	1.000	0.982
CLL/SLL	All	0.87 (0.68–1.11)	6	54.6%	0.051	0.707	0.796
MZL	All	0.70 (0.57–0.87)	3	5.5%	0.347	n/a	n/a
B-cell	All	0.84 (0.68–1.05)	8	73.4%	0.000.	0.902	0.724
	Exc. Kleinstern [42]	0.76 (0.68–0.85)	7	43.5%	0.101	1.000	0.661
T-cell	All	0.70 (0.48–1.01)	8	64.2%	0.007	1.000	0.607
***(b) Dietary intake***							
NHL	All	1.03 (0.90–1.19)	9	56.9%	0.018	0.466	0.084
	Cohort study	1.03 (0.90–1.17)	4	0.0%	0.583	n/a	n/a
	Case-control study	0.995 (0.76–1.30)	5	73.8%	0.004	n/a	n/a
DLBL	Ref. by 200IU/d	0.98 (0.81–1.20)	7	0.0%	0.550	n/a	n/a
	Ref. by max. intake	0.98 (0.80–1.21)	7	0.0%	0.547	n/a	n/a
FL	Ref. by 200IU/d	1.04 (0.81–1.33)	7	45.9%	0.085	n/a	n/a
	Ref. by max. intake	0.96 (0.74–1.24)	7	26.1%	0.229	n/a	n/a
CLL/SLL	Ref. by 200IU/d	0.95 (0.61–1.48)	6	57.3%	0.039	n/a	n/a
	Ref. by max. intake	0.99 (0.73–1.33)	6	41.4%	0.130	n/a	n/a
MZL	Ref. by 200IU/d	0.98 (0.54–1.77)	2	0.0%	0.612	n/a	n/a
	Ref. by max. intake	0.91 (0.49–1.70)	2	0.0%	0.730	n/a	n/a
T-cell	Ref. by 200IU/d	1.49 (0.84–2.66)	3	0.0%	0.642	n/a	n/a
	Ref. by max. intake	1.55 (0.83–2.88)	3	45.2%	0.161	n/a	n/a
***(c) Serum/plasma 25(OH)D***							
NHL	All	0.97 (0.82–1.15)	3	0.0%	0.543	n/a	n/a
	Exc. Łuczyńska [17]	1.01 (0.82–1.24)	2	0.0%	0.374	n/a	n/a
DLBL	All	1.01 (0.74–1.37)	2	0.0%	0.437	n/a	n/a
FL	All	1.20 (0.83–1.72)	2	0.0%	0.453	n/a	n/a
CLL/SLL	All	0.82 (0.62–1.10)	2	6.3%	0.302	n/a	n/a

Meta-analyses results are presented in summary relative risks (RR) and their 95% confidence intervals (95% CI). Kleinstern et al. [42] is a study with influence on heterogeneity; Łuczyńska et al., [17] is a study with outcome in B-cell NHL (not NHL) but was included in line with a previous meta-analysis.

Regarding dietary vitamin D intake, risk estimates were non-significant and showed inconsistent direction of associations for NHL (RR = 1.03, 95% CI: 0.90–1.19), and for subtypes (DLBL, RR = 0.98, 95% CI: 0.81–1.20; FL, RR = 1.04, 95% CI: 0.81–1.33; CLL/SLL, RR = 0.95, 95% CI: 0.61–1.48; MZL, RR = 0.98, 95% CI: 0.54–1.77; and T-cell NHL, RR = 1.49, 95% CI: 0.84–2.66) among subjects with intake over approximately 200 IU/day.

Although non-significant, risk estimates were greater than 1 among subjects with lower 25(OH)D level (<50 nmol/L; reference range 50–75 nmol/L) for NHL as well as its subtypes (NHL, RR = 1.01, 95% CI: 0.82–1.24; DLBL, RR = 1.01, 95% CI: 0.74–1.37; FL, RR = 1.20, 95% CI: 0.83–1.72; CLL/SLL, RR = 0.82, 95% CI: 0.62–1.10; reference range 50–75 nmol/L).

### Subgroup analysis by different sunlight/UVR exposure measures

Risk estimates for subgroups of sunlight/UVR exposure by various measures are shown in Table 3. Parallel to RRs for overall sunlight/UVR exposure on NHL and subtypes ranging from 0.70–0.87 (all tested outcomes significant except for T-cell NHL), statistically significant RRs with similar patterns of a protective effect were observed for sunburn (range 0.57–0.76; for all cases of NHL, DLBL, FL, CLL/SLL, and B-cell NHL), bathing vacation (range 0.65–0.78; DLBL and CLL/SLL), sunbath/suntan (range 0.60–0.63; FL and CLL/SLL), ambient exposure (range 0.55–0.90; FL, CLL/SLL, B-cell NHL, and T-cell NHL), outdoor activity/recreational activity (range 0.25–0.77; NHL and T-cell NHL), and composite total exposure (RR = 0.82; NHL). Exposure to artificial tanning was neither significantly associated with NHL nor any of the subtypes.

**Table 3:** Sunlight/UVR exposure in various measures and non-Hodgkin lymphoma risk.

		NHL	DLBL	FL	CLL/SLL	MZL	B-cell	T-cell
**Overall sunlight/UVR exposure**	Summary RR (95% CI)	0.80 (0.71–0.90)	0.73 (0.62–0.86)	0.77 (0.71–0.84)	0.87 (0.80–0.94)	0.70 (0.57–0.87)	0.76 (0.68–0.85)	0.70 (0.48–1.01)
	Study no. (I^2^)	17 (65.8%)	7 (57.4%)	8 (16.0%)	7 (45.5%)	3 (5.5%)	7 (43.5%)	8 (64.2%)
	p-heterogeneity	< .001	0.029	0.305	0.088	0.347	0.101	0.007
**(i) Sunburn**	Summary RR (95% CI)	0.72 (0.62–0.83)	0.57 (0.46–0.71)	0.74 (0.58–0.95)	0.65 (0.49–0.87)	0.91 (0.52–1.58)	0.76 (0.61–0.94)	0.72 (0.41–1.25)
	Study no. (I2)	5 (26.9%)	3 (48.8%)	3 (0.0%)	3 (0.0%)	2 (0.0%)	2 (0.0%)	2 (33.4%)
	p-heterogeneity	0.243	0.142	0.889	0.707	0.418	0.418	0.221
**(ii) Bathing vacation**	Summary RR (95% CI)	0.91 (0.59–1.40)	0.78 (0.61–0.99)	0.74 (0.45–1.20)	0.65 (0.52–0.81)	1.08 (0.30–3.93)	0.98 (0.38–2.57)	0.75 (0.54–1.05)
	Study no. (I^2^)	4 (88.0%)	3 (32.6%)	3 (50.4%)	3 (85.7%)	2 (54.0%)	2 (91.9%)	3 (0.0%)
	p-heterogeneity	< .001	0.227	0.133	0.001	0.140	< .001	0.603
**(iii) Artificial tanning**	Summary RR (95% CI)	0.92 (0.84–1.003)	0.83 (0.66–1.06)	0.92 (0.49–1.74)	1.001 (0.80–1.26)	-	0.95 (0.57–1.59)	1.23 (0.86–1.75)
	Study no. (I^2^)	7 (30.2%)	2 (0.0%)	2 (74.4%)	2 (0.0%)	-	2 (74.1%)	2 (0.0%)
	p-heterogeneity	0.198	0.39	0.048	0.98	-	0.049	0.822
**(iv) Sunbath/suntan**	Summary RR (95% CI)	0.78 (0.50–1.23)	0.64 (0.36–1.15)	0.60 (0.45–0.81)	0.63 (0.51–0.79)	1.24 (0.11–14.03)	0.94 (0.38–2.30)	0.89 (0.55–1.43)
	Study no. (I^2^)	4 (77.6%)	3 (70.1%)	3 (0.0%)	3 (34.0%)	2 (72.8%)	2 (86.8%)	3 (0.0%)
	p-heterogeneity	0.004	0.035	0.398	0.22	0.055	0.006	0.987
**(v) Ambient exposure (e.g. TOMS estimate)**	Summary RR (95% CI)	0.79 (0.67–0.92)	0.72 (0.51–1.01)	0.85 (0.72–1.02)	0.96 (0.76–1.19)	-	0.77 (0.64–0.94)	0.55 (0.40–0.75)
	Study no. (I^2^)	7 (66.8%)	3 (51.7%)	4 (0.0%)	4 (0.0%)	-	2 (0.0%)	3 (38.9%)
	p-heterogeneity	0.006	0.126	0.410	0.480	-	0.810	0.195
**(vi) Outdoor activity/recreational exposure**	Summary RR (95% CI)	0.77 (0.59–0.99)	1.30 (0.98–1.74)	1.24 (0.83–1.85)	-	-	1.09 (0.91–1.31)	0.25 (0.10–0.60)
	Study no. (I^2^)	2 (0.0%)	2 (0.0%)	2 (0.0%)	-	-	3 (49.5%)	2 (0.0%)
	p-heterogeneity	0.354	0.708	0.869	-	-	0.138	0.437
**(viii) Total exposure (composite or h/wk)**	Summary RR (95% CI)	0.82 (0.69–0.97)	-	-	-	-	-	-
	Study no. (I^2^)	4 (0.0%)	-	-	-	-	-	-
	p-heterogeneity	0.572	-	-	-	-	-	-

## Discussion

In this study, we investigated the association between vitamin D status and the risk of NHL incidence, and evaluated three different measures of vitamin D status over various NHL subtypes. The meta-analysis showed a statistically significant protective effect of sunlight/UVR exposure on NHL and its subtypes in most association results, in overall and in subgroups of different sunlight/UVR measure categories, although non-significant when only prospective studies were considered. These findings are consistent with those of previous reports [6–10]. Vitamin D status measured as dietary intake and 25(OH)D levels showed a non-significant relationship with NHL incidence, similar to a previous meta-analysis [13]. The summary RR for the effect of dietary intake was inconsistent across different subtypes, while a positive direction of risk estimates was observed for 25(OH)D levels, i.e., the RR among vitamin D-deficient subjects compared to non-deficient subjects was greater than 1.

A possible mechanism underlying the role of vitamin D in the incidence of NHL is explained by the expression of vitamin D receptors and 1α-hydrolase, an enzyme that converts the circulating form of vitamin D (25(OH)D) into the bioactive metabolite (1,25-dihydroxyvitamin D), in activated B-and T-lymphocytes, and thus by the autocrine and paracrine role of vitamin D in regulating cell proliferation as well as inducing apoptosis and differentiation [44–47].

The major natural source of vitamin D is through skin exposure to sunlight, specifically ultraviolet B radiation, and adequate amounts can be synthesized with sufficient exposure to sunlight [48]. On the other hand, vitamin D is not present in most foods, and is usually not in abundant amounts [49]. The different sources and contributions of vitamin D could explain the high prevalence of vitamin D deficiency in the modern world, especially in populations residing at high latitudes, retaining indoor-oriented lifestyle, or with cultures that do not prefer suntanned skin [5]. This implication could also explain the gap between different measures of vitamin D status in our meta-analysis. While the immediate interpretation of an exposure-outcome relationship is allowable on the risk estimates evaluated by direct exposure measures, i.e., sunlight/UVR, caution is needed with dietary intake of vitamin D as it covers a small proportion of the actual vitamin D exposure/status. Information collection by food frequency questionnaires subjectively answered by the study participants could have also contributed to some misclassification bias.

Our third vitamin D status measure, 25(OH)D level, is more complex. Although 25(OH)D levels could reflect the actual vitamin D status of an individual, there is much confounding due to variables such as age, sex, race/ethnicity, etc. Also, there has been much controversy on the assessment of the biomarker itself, especially regarding the inconsistent analysis quality. Consensus on the assay standardization was only arrived at in the 2010s [50–52]. Most of the studies evaluated in the current meta-analysis had adjusted for the known confounders, but a few used plasma levels and not serum levels. Moreover, 25(OH)D analyses in all studies were carried out from the 1970s to the 2000s, at the latest. Thus, controversies on the interpretation of the results of the association between 25(OH)D and NHL remain, and could be resolved by future studies using the 25(OH)D assay method with improved quality.

To our knowledge so far, this meta-analysis reviewed most recent relevant studies on vitamin D status and the risk of NHL incidence. The major strength of this meta-analysis lies in the collective evaluation of as many exposure measures and NHL subtypes as possible, which has not been previously attempted. Especially in the case of sunlight/UVR exposure, we attempted to show both the combined and separate measures, considering the vast diversity of the exposure categories. As a result, we were able to confirm the significant effect of naturally-occurring sunlight/UVR exposure, such as sunbath/suntan and bathing vacations. With the accumulating knowledge on NHL as a complex disease entity, it is very important to examine the risk by the subtypes. Thus, much effort was made to collect as much information as possible from the available studies, and we were able to evaluate risk estimates in subtypes with high incidence rates in both Caucasian and Asian populations [3]. Also, our consistent finding of the effect of sunlight/UVR exposure among different subtypes supported the latest report by the InterLymph Subtypes Project group that suggested that while subtype-specific mechanisms exist, statistically significant variability among the subtypes was not observed for sunlight exposure (p for heterogeneity = 0.79) [53]. Finally, we carried out several sensitivity analyses to evaluate heterogeneity or publication bias across the studies.

This meta-analysis has several limitations. First, most of the study subjects were non-Hispanic Caucasians in European or North America, and very few studies with different races/ethnicities were included. The authors had also tested the selection process including searching for non-English publications and found that the results were unchanged. Therefore, there could be limited generalizability of the results, as the incidence and distribution of NHL and specific subtypes vary by population or geographical region. Second, although optimal efforts were made to impartially combine or accurately classify the mixed and non-uniform categories of sunlight/UVR exposure measures, the overall and separate categories were arranged according to the subjective assumptions of the authors. Finally, as observational studies were used in the analysis, our results are not exempt from some selection and misclassification biases. Some of the case-control studies included in the analysis had hospital controls, and exposure information was ascertained by interviews or self-reported questionnaires in many cohort and case-control studies.

In summary, this meta-analysis of observational studies regarding vitamin D status and NHL showed that the association outcomes differed according to the measures of exposure assessed. A consistent protective effect of sunlight/UVR exposure on NHL and the various subtypes was observed, while inconsistent or non-significant association was found for dietary vitamin D intake and serum/plasma 25(OH)D levels. Further well-conducted case-control and prospective studies may be recommended for the two yet-controversial exposure measures.

## Supporting information

S1 Table Study quality assessment based on the Newcastle-Ottawa scale. (DOCX)

S2 Table Additional details on general characteristics of the studies on vitamin D status and non-Hodgkin lymphoma risk. (DOCX)

S3 Table Meta-regression analysis on sunlight/UVR radiation and non-Hodgkin lymphoma incidence according to study characteristics. (DOCX)

S4 Table PRISMA checklist. (DOCX)

S1 Fig Funnel plot for meta-analysis of the association between vitamin D status and non-Hodgkin lymphoma incidence by different sources of exposure. (a) overall sunlight/UVR exposure; (b) dietary vitamin D intake.
(TIF)

S2 Fig Meta-regression of non-Hodgkin lymphoma incidence according to year of study commencement. Each circle represents a study and indicates its weight in the analysis.
(TIF)
