# Supplementary material for: Vitamin D status and risk of non-Hodgkin lymphoma: An updated meta-analysis
Source: PLoS One. 2019 Apr 29;14(4):e0216284. doi: 10.1371/journal.pone.0216284 (PMC6488072; doi:10.1371/journal.pone.0216284)
Supplement: S1 Table — (DOCX) [file pone.0216284.s001.docx]

**S1 Table. Study quality assessment based on the Newcastle-Ottawa scale.**

| **Author** | **Ref. no.** | **Selection** | | | | **Comparability** | | **Outcome** | | | **Total** |
| --- | --- | --- | --- | --- | --- | --- | --- | --- | --- | --- | --- |
|  |  | **Case definition** | **Representativeness of cases** | **Selection of Controls** | **Definition of controls** | **Control for main factor** | **Controls for additional factor** | **Ascertainment** | **Same method** | **Non-response rate** |  |
| ***Cohort studies*** | | | | | | | | | | | |
| Erber et al., 2010 | [20] | 1 | 1 | 0 | 1 | 1 | 1 | 1 | 1 | 1 | 8 |
| Freedman et al., 2010 | [21] | 0 | 1 | 0 | 1 | 1 | 1 | 1 | 1 | 1 | 7 |
| Veierød et al., 2010 | [22] | 1 | 1 | 0 | 1 | 1 | 1 | 1 | 1 | 1 | 8 |
| Bertrand et al., 2011 | [23] | 0 | 0 | 0 | 1 | 1 | 1 | 1 | 1 | 1 | 6 |
| Chang et al., 2011 | [24] | 0 | 0 | 1 | 1 | 1 | 0 | 1 | 1 | 1 | 6 |
| Lin et al., 2012 | [25] | 1 | 1 | 0 | 1 | 1 | 1 | 1 | 1 | 1 | 8 |
| Zhang et al., 2013 | [26] | 0 | 1 | 0 | 1 | 1 | 1 | 1 | 1 | 1 | 7 |
| ***Case-control studies*** | | | | | | | | | | | |
| Hughes et al., 2004 | [27] | 1 | 1 | 1 | 1 | 1 | 1 | 1 | 1 | 0 | 8 |
| Smedby et al., 2005 | [28] | 1 | 1 | 1 | 1 | 1 | 1 | 1 | 1 | 0 | 8 |
| Chang et al., 2006 | [29] | 1 | 1 | 1 | 1 | 1 | 1 | 0 | 1 | 0 | 7 |
| Hartge et al., 2006 | [30] | 1 | 1 | 1 | 0 | 1 | 1 | 0 | 1 | 0 | 6 |
| Polesel et al., 2006 | [31] | 1 | 1 | 0 | 1 | 1 | 1 | 0 | 1 | 0 | 6 |
| Soni et al., 2007 | [32] | 1 | 1 | 1 | 1 | 1 | 0 | 0 | 1 | 1 | 7 |
| Weihkopf et al., 2007 | [33] | 1 | 1 | 1 | 1 | 1 | 1 | 0 | 1 | 0 | 7 |
| Zhang et al., 2007 | [34] | 1 | 1 | 1 | 0 | 1 | 1 | 0 | 1 | 0 | 6 |
| Boffetta et al., 2008 | [35] | 1 | 1 | 1 | 1 | 1 | 1 | 0 | 1 | 0 | 7 |
| Grandin et al., 2008 | [36] | 1 | 1 | 0 | 1 | 1 | 0 | 1 | 1 | 0 | 6 |
| Kricker et al., 2008 | [6] | 1 | 1 | 1 | 1 | 1 | 1 | 1 | 1 | 0 | 8 |
| Kelly et al., 2010 | [37] | 1 | 1 | 0 | 1 | 1 | 1 | 0 | 1 | 0 | 6 |
| Purdue et al., 2010 | [38] | 1 | 1 | 1 | 1 | 1 | 1 | 1 | 1 | 0 | 8 |
| Kelly et al., 2012 | [39] | 1 | 1 | 0 | 1 | 1 | 1 | 0 | 1 | 1 | 7 |
| Mikhak et al., 2012 | [40] | 1 | 1 | 1 | 1 | 1 | 1 | 0 | 1 | 0 | 7 |
| Wong et al., 2012 | [41] | 1 | 1 | 0 | 1 | 1 | 1 | 0 | 1 | 0 | 6 |
| Łuczyńska et al., 2013 | [17] | 1 | 1 | 1 | 1 | 1 | 1 | 1 | 1 | 1 | 9 |
| Cerhan et al., 2014 | [7] | 1 | 1 | 1 | 1 | 1 | 1 | 1 | 1 | 0 | 8 |
| Linet et al., 2014 | [8] | 1 | 1 | 1 | 1 | 1 | 1 | 1 | 1 | 0 | 8 |
| Slager et al., 2014 | [9] | 1 | 1 | 1 | 1 | 1 | 1 | 1 | 1 | 0 | 8 |
| Bracci PM et al., 2014 | [10] | 1 | 1 | 1 | 1 | 1 | 1 | 1 | 1 | 0 | 8 |
| Kleinstern et al., 2017 | [42] | 1 | 1 | 0 | 1 | 1 | 1 | 0 | 1 | 0 | 6 |
| Wang et al., 2017 | [43] | 0 | 1 | 1 | 1 | 1 | 1 | 0 | 1 | 1 | 7 |
