# Supplementary material for: Vitamin D status and risk of non-Hodgkin lymphoma: An updated meta-analysis
Source: PLoS One. 2019 Apr 29;14(4):e0216284. doi: 10.1371/journal.pone.0216284 (PMC6488072; doi:10.1371/journal.pone.0216284)
Supplement: S2 Table — (DOCX) [file pone.0216284.s002.docx]

**S2 Table. Additional details on general characteristics of the studies on vitamin D status and non-Hodgkin lymphoma risk.**

| **Author** | **Ref. no.** | **Location** | **Sex** | **Race/ethnicity** | **Population type** | **Age** | **F/U years** | **Outcome measure** |
| --- | --- | --- | --- | --- | --- | --- | --- | --- |
| Erber et al., 2010 | [20] | Hawaii, Los Angeles | M&F | Caucasian, African American, Native Hawaiian, Japaniese American, Latino | general | 45-75 | 10 (med.) | HR |
| Freedman et al., 2010 | [21] | (USA) | M&F | Caucasian, mostly | radiation technologist | unspecified (m/c: <45) | 22 (max.) | OR |
| Veierød et al., 2010 | [22] | Sweden-UppsaLos Angeles Health Care Region | F | Caucasian | general | 30-50 | 15 (mean) | RR |
| Bertrand et al., 2011 | [23] | California, Connecticut, Florida, MaryLos Angelesnd, Massachusetts, Michigan, New Jersey, New York, Ohio, Pennsylvania, Texas | F | Caucasian, mostly | nurses | 30-55 | 30 (max.) | RR |
| Chang et al., 2011 | [24] | California | F | Caucasian >85% | teachers | 22-104 (med. 53) | 12 (max. | RR |
| Lin et al., 2012 | [25] | California, Florida, Lousiana, New Jersey, North Carolina, Pennsylvania; AtLos Angelesnta, Detroit | M&F | Non-Hispanic white | general | 50-71 | 9 (mean) | HR |
| Zhang et al., 2013 | [26] | (USA) | F | Caucasian, mostly | nurses | 25-42 | 20 (max.) | HR |
| Hughes et al., 2004 | [27] | New South Wales, Australian Capital Territory | M&F | Caucasian, mostly | general | 20-74 | - | OR |
| Smedby et al., 2005 | [28] | (Denmark, Sweden) | M&F | Caucasian | general | 18-74 (med. 61) | - | OR |
| Chang et al., 2006 | [29] | (Sweden) | M&F | Caucasian | general | 18-74 | - | OR |
| Hartge et al., 2006 | [30] | Iowa, Los Angeles; Detroit, Seattle | M&F | Caucasian, mostly | general | 20-74 | - | RR |
| Polesel et al., 2006 | [31] | Pordenone(N.Italy), Naples(S.Italy) | M&F | Caucasian, mostly | general (hospital control) | 18-84 | - | OR |
| Soni et al., 2007 | [32] | Nebraska | M&F | Caucasian >95% | general | 20-75 | - | OR |
| Weihkopf et al., 2007 | [33] | Ludwigshafen/Upper PaLos Angelestinate, Heidelberg/Rhine-Neckar Country, Wurzburg/Lower Frankonia, Hamburg, Bielefeld/Gutersloh, Munich | M&F | Caucasian, mostly | general | 18-80 | - | OR |
| Zhang et al., 2007 | [34] | Connecticut | F | Caucasian, mostly | general | 21-84 | - | OR |
| Boffetta et al., 2008 | [35] | (21 centers) | M&F | Caucasian | general (population & hospital control) | 18-89 (med. 62) | - | OR |
| Grandin et al., 2008 | [36] | Bordeux, Brest, Caen, Lille, Nantes, Tolulouse | M&F | Caucasian | general (hospital control) | 20-75 | - | OR |
| Kricker et al., 2008 | [6] | BC(00-04), Mayo Clinic(02-05), NCI-SEER(98-00), Nebraska(99-02); EpiLymph1&2 (98-04), SCALE(00-02), UK(98-01); NSW(00-02) | M&F | Caucasian, mostly | general (population & hospital control) | 16-96 (med. 59) | - | OR |
| Kelly et al., 2010 | [37] | New York | M&F | Caucasian, mostly | general (hospital control) | ≥21 | - | OR |
| Purdue et al., 2010 | [38] | ATBC(85-88), CPS-II(98-01), CLUE I&II(Washington County, Marlyand, 74&89), HPFS(93-95), MEC(Hawaii & California, 01-06), NYU-WHS(NY, 85-91), NHS(89-90), PLCO(93-01); SMHS(Shanghai, 01-06), SWHS(97-00) | M&F | Caucasian 93% | general, mostly (HPFS-health professional, ATBC-smoker, NYU-WHS-mammography screenee, NHS-nurse) | 49-75 (IQR range) | - | OR |
| Kelly et al., 2012 | [39] | Iowa, Minnesota, Wisconsin | M&F | Caucasian, mostly | general (hospital control) | ≥20 | - | OR |
| Mikhak et al., 2012 | [40] | San Francisco | M&F | Caucasian, mostly | general | 20-85 | - | OR |
| Wong et al., 2012 | [41] | (Singapore) | M&F | Asian (Chinese 77%, Malay 14%, Indian 8%) | general (hospital control) | 18-87 (mean 50.3) | - | OR |
| Łuczyńska et al., 2013 | [17] | [Core] Spain(Grananda, Murcia, Navarra, Asturias); Italy(Ragusa, Florence, Turin, Varese); Germany(Heidelberg, Potsdam); NetherLos Angelesnds(Bilthoven, Utrecht); UK(Cambridge, Oxford); [Associated] Italy(Naples), Denmark(Aarhus), Sweden(Malmo, Umea), Norway(Tromso) | M&F | Caucasian, mostly | general (population & hospital control) | 35-65 | - | IRR (RR) |
| Cerhan et al., 2014 | [7] | BC(Vancouver, Victoria, BC, 00-04), Iowa/Minnesota(81-83), Kansas(76-82), Los Angeles(89-92), Mayo Clinic(Iowa, Minnesota, Wisconsin, 02-08), NCI-SEER(Detroit, Michigan, Iowa, Los Angeles, California, Seattle, Washington, 98-00), Nebraska(83-86, 99-02), UCSF1&2(San Francisco, 88-95, 01-06), Univ. Rochester(NY, 05-07), Yale(CT, 95-01); EngeLos Angeles(00-04), EpiLymph, Italy multicenter(Firenze, Forli, Imperia, Los Angelestina, Novara, Ragusa, Siena, Torino, Varese, Vercelli, Verona, 90-93), Italy (Aviano-MiLos Angelesn, 83-92), Italy (Aviano-Naples, 99-02), SCALE(99-02), UK(Los Angelesncashire/ S.Los AngeleskeLos Angelesnd, S.EngLos Angelesnd, Yorkshire, 98-01); NSW(00-01) | M&F | Caucasian 93% | general (population & hospital control) | unspecified (m/cr: 60-69) | - | OR |
| Linet et al., 2014 | [8] |  | M&F | Caucasian 93% | general (population & hospital control) | 18-91 (med. 58) | - | OR |
| Slager et al., 2014 | [9] |  | M&F | Caucasian 93% | general (population & hospital control) | 28-93 (med. 64) | - | OR |
| Bracci PM et al., 2014 | [10] |  | M&F | Caucasian 93% | general (population & hospital control) | unspecified (m/c: 60-69) | - | OR |
| Kleinstern et al., 2017 | [42] | (Israel. Palestine) | M&F | Caucasian (Jew/Arab) | general | ≥18 | - | OR |
| Wang et al., 2017 | [43] | Los Angeles | F | Non-Hispanic white | general | 20-79 | - | OR |
