# Supplementary material for: Vitamin D status and risk of non-Hodgkin lymphoma: An updated meta-analysis
Source: PLoS One. 2019 Apr 29;14(4):e0216284. doi: 10.1371/journal.pone.0216284 (PMC6488072; doi:10.1371/journal.pone.0216284)
Supplement: S3 Table — (DOCX) [file pone.0216284.s003.docx]

**S3 Table. Meta-regression analysis on** **sunlight/UVR radiation and non-Hodgkin lymphoma incidence according to study characteristics.**

| **Factor** | **Summary RR (95% CI)** | **P-value** | **I^2^** |
| --- | --- | --- | --- |
| NOS | 0.95 (0.8-1.12) | 0.517 | 64.09 |
| Study design | 0.85 (0.65-1.11) | 0.222 | 60.26 |
| Race/ethnicity | 0.87 (0.48-1.59) | 0.628 | 67.16 |
| Year of study commencement | 0.98 (0.97-0.995) | 0.012 | 43.07 |
| Year of publication | 1.02 (0.97-1.08) | 0.33 | 61.81 |
| No. of cases | 0.99999 (0.99992-1.0001) | 0.703 | 65.79 |
| No. of study population | 1.0000003 (0.999999-1.0000014) | 0.637 | 67.75 |
