# Supplementary figures and images for: Vitamin D status and risk of non-Hodgkin lymphoma: An updated meta-analysis
Source: PLoS One. 2019 Apr 29;14(4):e0216284. doi: 10.1371/journal.pone.0216284 (PMC6488072; doi:10.1371/journal.pone.0216284)

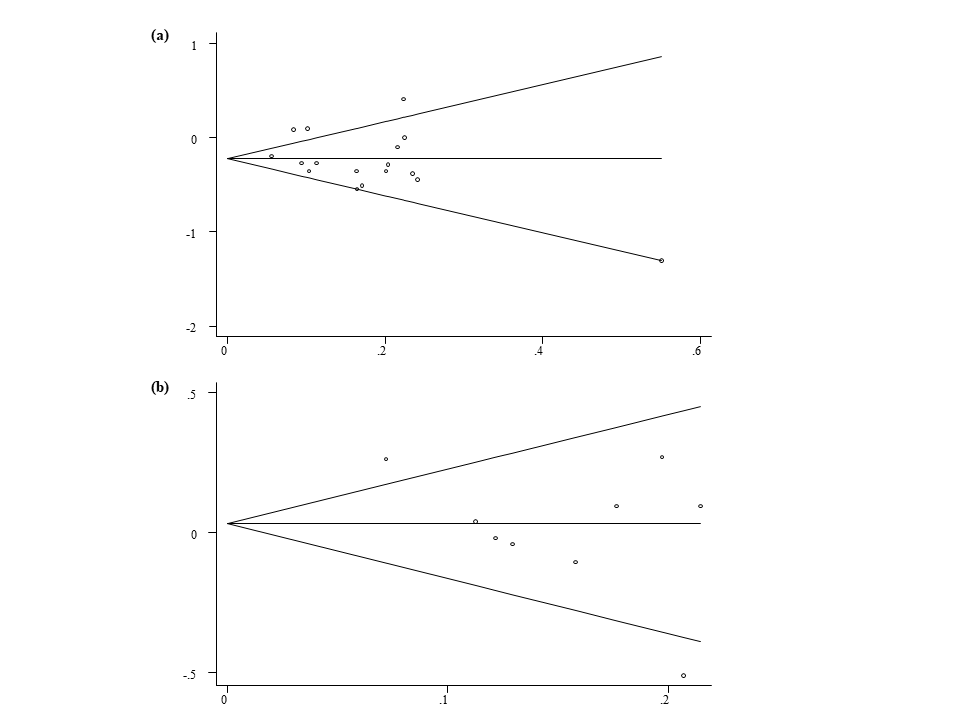

Supplement: S1 Fig — (a) overall sunlight/UVR exposure; (b) dietary vitamin D intake. (TIF) [file pone.0216284.s005.tif]

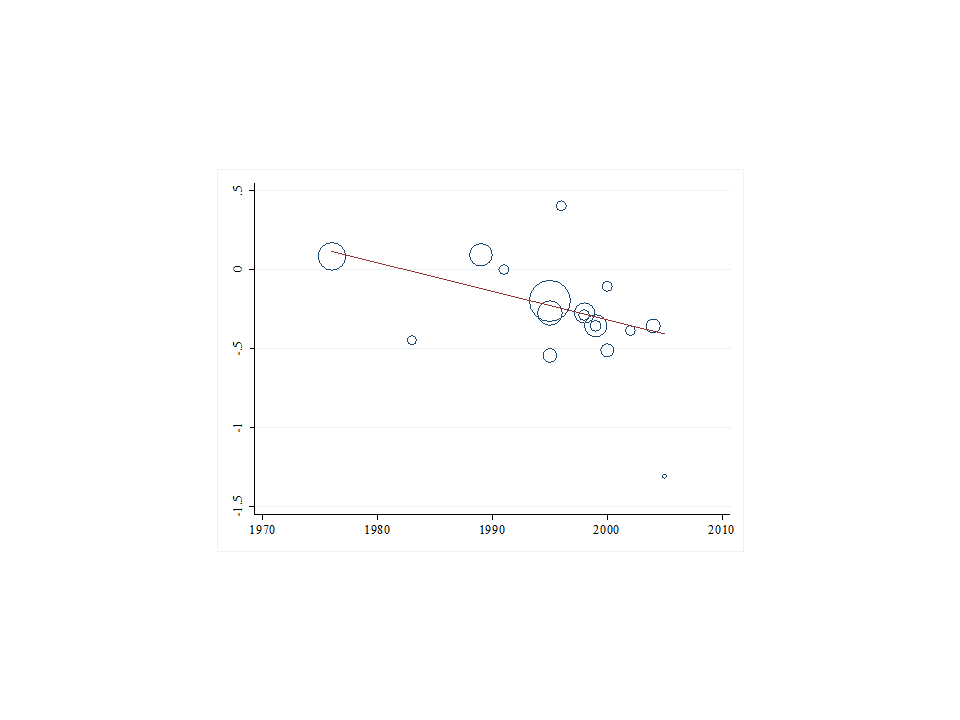

Supplement: S2 Fig — Each circle represents a study and indicates its weight in the analysis. (TIF) [file pone.0216284.s006.TIF]
